# Mitochondria: great balls of fire

**DOI:** 10.1111/febs.17316

**Published:** 2024-11-14

**Authors:** Howard T. Jacobs, Pierre Rustin, Paule Bénit, Dan Davidi, Mügen Terzioglu

**Affiliations:** ^1^ Faculty of Medicine and Health Technology Tampere University Finland; ^2^ Department of Environment and Genetics La Trobe University Melbourne Australia; ^3^ Université Paris Cité, Inserm, Maladies neurodéveloppementales et neurovasculaires Paris France; ^4^ Department of Genetics Harvard Medical School Boston MA USA; ^5^ Present address: Aleph Tel Aviv‐Yafo Israel

**Keywords:** cold‐shock, eukaryote origins, heat‐shock, homeothermy, immunity, mitochondria, mitochondrial disease, mitochondrial dynamics, temperature gradients, thermogenesis

## Abstract

Recent experimental studies indicate that mitochondria in mammalian cells are maintained at temperatures of at least 50 °C. While acknowledging the limitations of current experimental methods and their interpretation, we here consider the ramifications of this finding for cellular functions and for evolution. We consider whether mitochondria as heat‐producing organelles had a role in the origin of eukaryotes and in the emergence of homeotherms. The homeostatic responses of mitochondrial temperature to externally applied heat imply the existence of a molecular heat‐sensing system in mitochondria. While current findings indicate high temperatures for the innermost compartments of mitochondria, those of the mitochondrial surface and of the immediately surrounding cytosol remain to be determined. We ask whether some aspects of mitochondrial dynamics and motility could reflect changes in the supply and demand for mitochondrial heat, and whether mitochondrial heat production could be a factor in diseases and immunity.

## Graphical abstract

What are the implications of hot mitochondria? Recent experimental studies indicate that mitochondria in mammalian cells are maintained at temperatures of at least 50 °C. Here, we consider the ramifications of this finding for cellular functions, evolution, and disease.

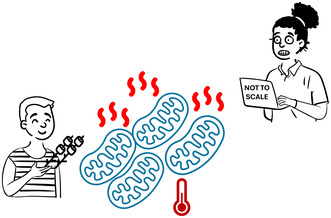

**Abbreviations**
AOX: alternative oxidase
ATP: adenosine triphosphate
cI: OXPHOS (or respiratory) complex I
cIII: OXPHOS (or respiratory) complex III
cIV: OXPHOS (or respiratory) complex IV
cV: OXPHOS complex V
EMBO: European Molecular Biology Organization
ER: endoplasmic reticulum
FCCP: carbonyl cyanide‐*p*‐trifluoromethoxyphenylhydrazone
GETI: genetically encoded temperature indicator
HFSP: Human Frontier Science Program
IMM: inner mitochondrial membrane
IMS: mitochondrial inter‐membrane space
MAMs: mitochondria‐associated membranes (of the ER)
MTY: Mito Thermo Yellow
NK (cells): natural killer (cells)
OMM: outer mitochondrial membrane
OXPHOS: oxidative phosphorylation
TCA (cycle): tricarboxylic acid (cycle)
*T*_m_
: melting temperature, the temperature at which half of the amount of a macromolecule is denatured
UCP1: uncoupling protein 1
UV: ultra‐violet

## Introduction

In two recent papers [1, 2], we presented data indicating that the temperature of probes targeted to mitochondria is some 15 °C warmer than the extracellular environment, both in mammalian and insect cells. Furthermore, mitochondrial temperature appeared to be largely resistant to metabolic stresses such as nutrient deprivation, altered ATP demand or even OXPHOS toxins if alternative metabolic pathways are available. This property indicates the existence of an unknown machinery to modulate mitochondrial activity in response to such stresses, so that intramitochondrial temperature is maintained, where physiologically possible, within narrow limits.

The meaning of physical laws governing thermal conductivity at the molecular/nano scale is debatable. At nano scale, the thermal conductivity predicted by Fourier's law is no longer an intrinsic property of materials, resulting in unexpected heat dissipation behavior [3]. However, theory and experimental data can also be reconciled [4]. Importantly, two independent methods—the temperature‐sensitive dye MTY and the fluorescence ratio between two co‐expressed reporter proteins with different temperature response profile, denoted gTEMP or GETI for genetically encoded temperature indicator—generate similar estimates for mitochondrial temperature. Moreover, a confounding influence from a variety of chemical parameters, such as pH, calcium ion concentration, membrane potential and reactive oxygen species has been excluded, at least in the case of MTY [1, 2]. Obviously this does not rule out an effect of an unknown variable to which the probes might respond, e.g., water potential [5] or magnetic field [6]. However, for simplicity, we shall assume here that temperature differences alone underlie the observed fluorescence changes and that mitochondria or, more precisely, the intramitochondrial compartment(s) into which these temperature‐sensitive probes are concentrated are, indeed, some 15 °C warmer than the extracellular environment, at least under standard culture conditions. Furthermore, since the fluorescence readings reach and then maintain a plateau level, we take this as an equilibrium value, i.e., reflecting a balance between heat production by the molecular machinery of oxidative phosphorylation (OXPHOS) in the inner mitochondrial membrane (IMM) and its dissipation to the rest of the cell. Although obviously oversimplified, it is perhaps useful to consider the analogy of a car propelled by an internal combustion engine. Fuel, equivalent to a biological substrate, is burned to produce useful energy—kinetic motion of the car, or ATP in the case of mitochondria. However, since the process is far from being 100% efficient thermodynamically, a variable but potentially substantial proportion of the released energy is converted to heat. Once the engine reaches an equilibrium operating temperature, the heat generated is balanced by heat loss via a cooling system, which can be regulated by the operator according to external conditions, in order to prevent over‐heating. The same principle applied to mitochondria implies that heat generation, influenced by both the rate and efficiency of substrate oxidation, is balanced against heat dissipation to the cytosol and beyond, and that these process can all be adjusted by homeostatic mechanisms that remain largely unknown.

The technical limitations of current methods mean that the published findings on mitochondrial temperature should be treated with caution. Future experiments will need to address the outstanding technical issues, including the relationship between probe fluorescence and respiratory control, as well as the nature of the molecular machinery that monitors and regulates heat production and dissipation within the cell. Nevertheless, we here review the issues raised by the most straightforward interpretation of the recent findings and suggest how they might be addressed by future experimentation. We first address evolutionary and environmental considerations of elevated mitochondrial temperature, followed by the implications for cellular processes and, finally, the medical dimension.

## Evolutionary issues

Dunn [7] has put forward the hypothesis that one of the drivers of the ancient cellular symbiosis that led to the emergence of the first eukaryotes was that the mitochondrial ancestor was heat‐producing, while its archaeal host arose in and was adapted to a hot environment, e.g., in the vicinity of oceanic hydrothermal vents. Aerobic bacteria, having evolved an efficient way of energizing biological processes at lower temperatures, would also be releasing a great deal of heat to their environment. Thus, by associating intimately with these ‘mobile heaters’, ancient thermophilic archaea would have acquired the ability to migrate to and survive in cooler environments. In time, this would have enabled all of the metabolic and developmental processes that characterize eukaryotes, gradually adapting to life at an intermediate intracellular temperature.

There remains no clarity concerning whether the original symbiosis arose in an aerobic or anaerobic environment, and whether it occurred before or after the evolution of oxygenic photosynthesis. However, most of the mitochondria‐related organelles found in present‐day anaerobes are considered to have been secondary innovations [8]. There remain a number of hypothetical scenarios for how and when the endosymbiotic association began and became permanent. Dunn's idea has the virtue that it doesn't depend on an exchange of ATP or any other metabolite, as the basis for the initial association between host and symbiont. It would also be consistent with the initial symbiosis being extracellular, with engulfment of the symbiont as a subsequent step. In addition, it is consistent with the fact that the adenine nucleotide carrier, as well as the rest of the mitochondrial carrier family to which it belongs, appears to be a uniquely eukaryotic innovation. Heat exchange may thus have predated metabolic exchange. The recent inference that the Asgard archaeal ancestor of eukaryotes was a moderate thermophile living at the same temperature as present‐day mammalian mitochondria [9] gives further weight to the hypothesis that eukaryogenesis was driven by the heat‐supplying function of the ancestral endosymbiont that later evolved into mitochondria.

## Homeothermy

A particularly intriguing side issue is the question of why mammals and birds, considered the only true homeothermic organisms, maintain their body temperature around 40 °C except under some specific adaptive conditions, such as hibernation. Indeed, mammals appear to be highly sensitive to heat‐shock and cannot survive prolonged body temperatures above ~ 45 °C. This uniformity of body temperature applies to animals of enormously different sizes and external architecture, indicating that evolutionary adaptation to temperature extremes is much more easily achieved by a small number of body modifications than by a uniform shift in the temperature optima of all of the proteins encoded in the genome. The question remains as to what is so special about 40 °C. It seems reasonable to assume that this temperature represents a trade‐off between the rate of heat production by mitochondria and its loss to the environment. Logically, given the long‐term drop in ocean temperature over geological time [10], it would reflect a period in which this parameter had reached below 40 °C. This period [11] corresponds with the time of emergence of vertebrates living predominantly or exclusively on land, around 350–300 million years before present, after which the homeotherms soon arose. Nevertheless, if homeothermy is so advantageous, why did it not evolve in plants, fungi, and the many terrestrial invertebrates, all of which evolved and thrived on land for hundreds of millions of years before birds and mammals emerged?

Our observation that the mitochondria of *Drosophila* cells cultured at 25 °C are also at least ~ 15 °C warmer indicates that there is nothing special about the mammalian mitochondrial temperature of ~ 52 °C, rather that the biochemistry and architecture of mitochondria—and of the eukaryotic cell as a whole—imposes a rather constant temperature difference from the cytosolic and external environment. However, it should prompt a rethink of the way we use nomenclature: poikilotherms are not exotherms. Much of their heat is generated internally in mitochondria, and their cells may therefore be warmer than their external environment under most conditions. This surely also applies to plants, where considerable heat must also be generated in chloroplasts, although chloroplast temperature has not yet been directly studied.

## Thermogenesis

Mitochondrial thermogenesis in brown adipose tissue via the uncoupling protein UCP1 is a well‐established mechanism for cold‐adaptation in mammals and has been extensively reviewed elsewhere [12]. However, our findings on mitochondrial temperature highlight the fact that mitochondrial heat production is an inescapable aspect of aerobic metabolism, applicable to all tissues and organisms. Plants are also endowed with several families of thermogenic mitochondrial proteins [13] that effectively uncouple OXPHOS under defined conditions. In specific taxa, this process enables heating of reproductive organs, mainly made up of cells with tightly packed mitochondria, to temperatures up to 20 °C above ambient. This heating leads to the volatilization of insect attractants. A similar process underlies thermogenic responses to biotic and abiotic stresses, most likely mediated via the hormone salicylic acid [14, 15, 16]. A possible converse example, where excessive mitochondrial heat production could interfere with physiological processes, is suggested by the finding that exogenous expression of the thermogenic alternative oxidase in the testis tubule sheath disrupts spermatogenesis in *Drosophila* [17].

A cellular cooling effect from shutdown of OXPHOS or a warming effect from activation of the alternative oxidase or uncoupler proteins, both of which are widespread in eukaryotes, may have been overlooked as an explanation for many physiological processes and stress responses in nature. To give several possible examples, the high rate of protein synthesis in early embryos of the Antarctic sea urchin, *Sterechinus neumayerii*, comparable with that recorded in closely related species living in temperate or semi‐tropical waters [18], might be attributable to a localized intracellular warming. The evolution of homeothermy has been suggested to reflect an increased metabolic flexibility of mitochondria as an adaptive mechanism, initially facilitating survival in the cold [19]. This concept might also explain the success of species able to survive in very different thermal environments, such as the invasive tunicate *Botryllus schlosseri* [20]. Genetic and epigenetic differences affecting metabolism are prominent population indicators in this species and correlate with minimum annual sea‐surface temperature [21]. Mitochondrial bioenergetic differences also distinguish thermotolerant Atlantic cod from the less adaptable Polar variety [22]. Intracellular temperature probes can now be deployed to address a great variety of biological issues, wherein localized, temporary or even sustained heat delivery is hypothesized to underlie adaptive responses.

## Mitochondrial temperature homeostasis

At the organellar level, our recent work revealed cases suggestive of metabolic remodeling that maintains mitochondrial temperature despite changes in external conditions [2]. For example, in mammalian cells expressing the alternative oxidase from *Ciona intestinalis*, which provides a thermogenic by‐pass of OXPHOS complexes III and IV (cIII, cIV), the initial decrease in mitochondrial temperature produced by inhibitors of these complexes is much less than in control cells [2]. Importantly, the initial cooling effect is gradually reversed, a phenomenon seen even when OXPHOS complex I (cI) is inhibited [2]. These findings indicate that electron flow is re‐routed through CoQ‐linked pathways to recover the basal mitochondrial temperature, even though this may lead to sub‐optimal mitochondrial ATP production. Similarly, when cytosolic protein synthesis is inhibited, resulting in a large decrease in cellular ATP demand, mitochondrial temperature fluctuations are minimal [2].

**Fig. 1:**
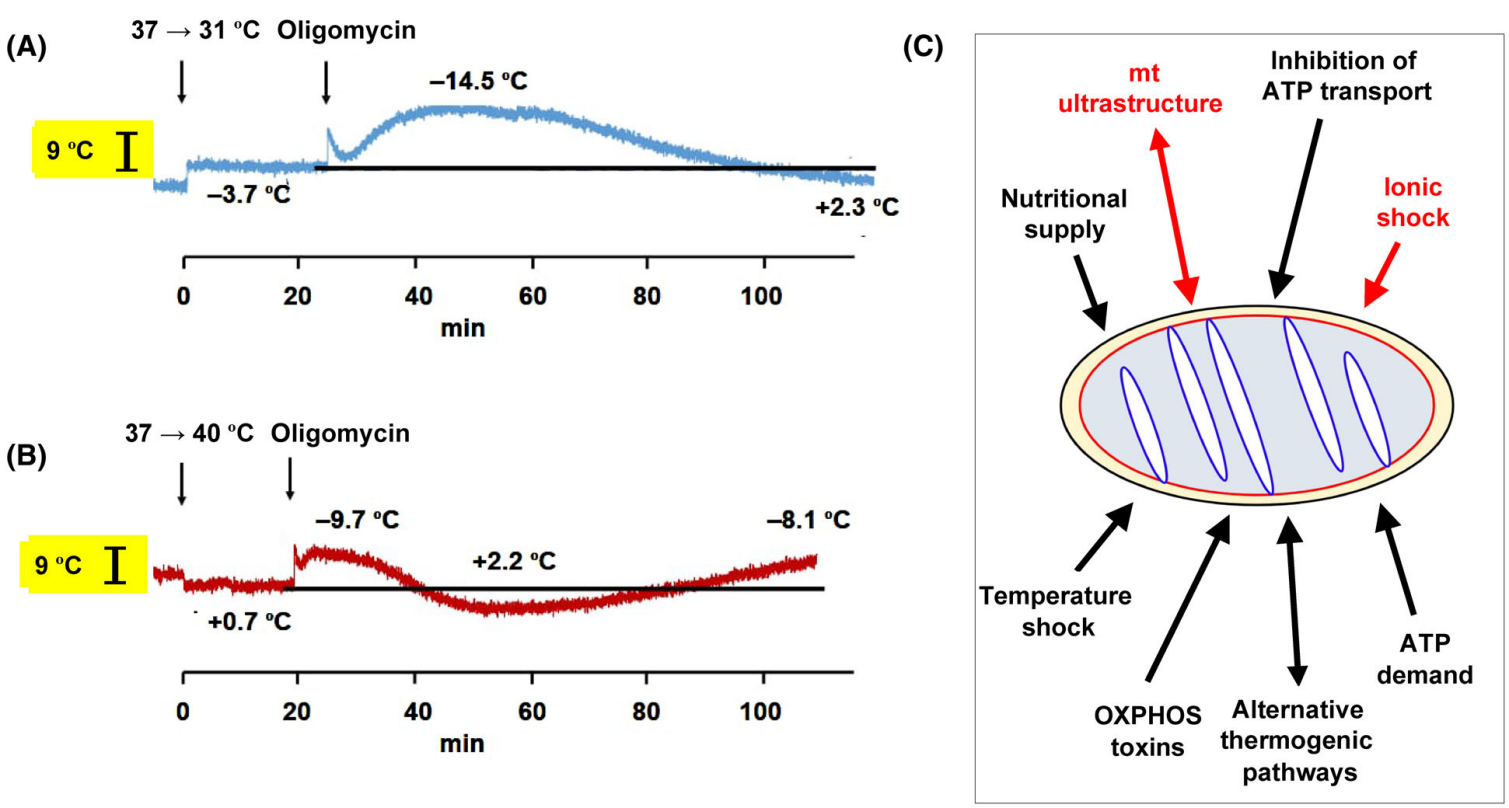
Stresses influencing mitochondrial temperature and heat production. (A, B) MTY (Mito Thermo Yellow) fluorescence traces (arbitrary values) indicating mitochondrial temperature changes resulting from (A) cold shock (37 → 31 °C) and (B) heat shock (37 → 40 °C), applied at time *t* = 0 (first vertical arrow) to cultured mouse embryo fibroblasts. Oligomycin was added to 5 μm at the indicated times (second vertical arrow). Same materials, methods, and calibration steps as described by Terzioglu *et al*. [2]. Shown are representative MTY fluorescence traces, annotated to indicate mean temperature shifts based on (A) 5 and (B) 3 independent experiments. Note that increased fluorescence indicates cooling and vice versa. Calibration values for the illustrated experiments are shown as black bars against yellow background. For source data, see Table S1. (C) Summary of stresses known [2] (in black) or hypothesized (in red) to induce homeostatic responses of mitochondrial temperature. Double‐headed arrows indicate processes assumed to operate in both directions. Black line: OMM (outer mitochondrial membrane), red line and blue line: respectively, inner boundary membrane and cristal membrane, considered together as IMM (inner mitochondrial membrane), cream oval: IMS (mitochondrial inter‐membrane space), pale blue oval: mitochondrial matrix.

To extend these findings, we have looked at the effect on mitochondrial temperature of altering the external temperature to which cells are subjected. Although the findings, illustrated in Fig. 1, are still preliminary, there again appears to be a homeostatic process at work, whereby mitochondrial heat production and/or dissipation is adjusted to the conditions, without long‐lasting disturbance of intramitochondrial temperature. Thus, when subjected to a 6 °C cold‐shock, dye fluorescence initially indicates a slightly lesser temperature decrease than the full 6 °C (Fig. 1A), possibly influenced by abrupt/transient changes in respiratory electron flow and/or probe distribution, but likely attributable to some thermal buffering between intracellular compartments. The further mitochondrial cooling produced by oligomycin inhibition of OXPHOS complex V (cV) is initially similar to that seen in cells maintained at 37 °C, both in its extent and kinetics. However, even in the presence of the drug, mitochondria appear to warm up again, reaching close to their initial temperature after 2 h of exposure to the drug, during which time the cells are maintained at 31 °C. Although further investigation is needed, in order to determine how other bioenergetic parameters change during this stress, the finding suggests that some thermogenic pathway independent of cV must be invoked. This could indicate a partial uncoupling of OXPHOS, or the activation of some other process not related to respiration. In heat‐shocked cells, the situation appears even more complex, based on similar preliminary data (Fig. 1B). Here too, the initial effect, namely warming, is partially buffered. The oligomycin‐induced mitochondrial cooling is less than in control cells, suggesting that OXPHOS has been partially disabled to compensate for the additional heat delivered externally. It declines further with time. However, prolonged exposure to oligomycin leads to a further progressive cooling of the mitochondria. Although preliminary, these findings indicate a further dimension of homeostatic temperature control inside mitochondria.

Mitochondrial temperature and heat production appear to respond to different stresses in ways that preserve mitochondrial integrity and function, but must also serve the physiological needs of the cell and the whole organism. As summarized in Fig. 1C, the stresses that induce thermostatic responses in mitochondria are diverse, while some remain to be tested, for example, ionic or osmotic shock, which may alter mitochondrial permeability properties, resulting in apoptosis if not rapidly reversed [23]. One obvious consequence of a loss of mitochondrial membrane potential is unrestrained respiratory electron flow, likely to entrain catastrophic overheating and a complete loss of structural integrity. However, a specific role of overheating in apoptotic signaling remains speculative. Osmotic stress may be considered more generally as a modulator of water potential [5], which is also influenced by alterations in protein folding mediated by specific ionic, metabolite and pH changes, as well as by the expression of chaperones.

The fact that mitochondria are sensitive *in situ* to an externally applied heat stress [24] highlights the importance of the need for mitochondria to respond to metabolic challenges by modulating their heat production, thus keeping their own temperature within physiologically tolerable limits, albeit that these remain unknown. The implied remodeling of metabolism in AOX‐expressing cells treated with OXPHOS toxins suggests that the maintenance of mitochondrial temperature—or its restoration following a disturbance—takes precedence over other aspects of metabolite homeostasis and even ATP production. In cells not endowed with AOX we envisage that other thermogenic pathways may be induced to buffer stresses arising from OXPHOS disruption. For example, after prolonged treatment with a cytosolic protein synthesis inhibitor, the maintenance of mitochondrial temperature is significantly less dependent on cI, but remains fully dependent on cV [2]. Although the underlying response mechanisms may be complex, the result implies a shift towards thermogenic ATP hydrolysis, thus maintaining mitochondrial temperature despite decreased respiratory flux. In AOX‐endowed cells, the opposite applies, with mitochondrial temperature maintenance more dependent on cI than on cV [2].

The availability of fluorescent temperature‐sensitive probes targeted to mitochondria, as well as to other subcellular compartments, now enables a much more exhaustive study of how mitochondrial temperature and heat production respond to a great variety of externally applied metabolic stresses.

## Temperature sensing

The narrow operating temperature range of mammalian and avian cells implies that mitochondrial heat production must be tightly regulated, so as to maintain adequate but not excessive heat supply under varying conditions. Although some classic second messengers, such as calcium ion, ROS and cyclic nucleotides may be involved in this regulation, we posit that there must exist a temperature sensing mechanism in the cytosol able to signal temperature changes or anomalies to mitochondria independently of other parameters.

The components of such a signaling system are unknown and may involve several spatially and temporally separate layers of control, as implied by the heat‐shock experiments described above. Logically, components of such a system, including sensors, transducers and effectors, must act locally, since different regions of a single cell may overheat or cool separately; such regulation is likely also to be tissue‐specific and should be capable of responding to emergency situations, e.g., by uncoupling OXPHOS or allowing for a more global shutdown of metabolism. There should also be sensors of temperature internal to the mitochondria, since over‐heating or ‐cooling of the organelle would risk permanently crippling its integrity and function. However, the mitochondrial and cytosolic systems of temperature sensing may not be entirely separate. If embedded in the outer mitochondrial membrane, a sensing system should be able to detect both a local anomaly in cytosolic temperature or in mitochondrial heat output/dissipation. There is precedent for the outer mitochondrial membrane playing a vital role in other aspects of cellular homeostasis, notably in the integration of life‐death signals, in the regulation of mitochondrial fusion, fission and motility, in the signals that control mitophagy and in the orchestration of antiviral and other immune responses. A mitochondrial temperature sensor embedded in the outer membrane could also be linked to any or all of these regulators, since they are likely to function in an interdependent manner. In the absence of any direct evidence for its existence or nature, we can only speculate.

The ability to sense the temperature of the external environment and adjust heat output accordingly must have been crucial for survival in the aerobic bacteria that were ancestral to mitochondria, as well as in their free‐living descendants today. We thus predict that the envisaged mitochondrial temperature sensor should have a homolog in the outermost membrane of today's bacteria, including those that have adopted an endosymbiotic lifestyle. The principal mechanism of temperature sensing in bacteria appears to be vested in the fluidity of the lipid bilayer [25] bounding the cell. Most work to characterize the molecular machinery involved has been carried out on the gram‐positive *Bacillus subtilis*, which is phylogenetically distant from the alpha‐proteobacteria to which the ancestral endosymbiont has been inferred to belong. Nevertheless, the likely universality of the mechanism means that clear homologs are likely to exist in alpha‐proteobacteria as well as other gram‐negatives, such as cyanobacteria. In addition, noting that the lipid composition of all eukaryotic membranes is of the bacterial rather than the archaeal type, it seems plausible that a homolog of the mitochondrial temperature sensor would be present also in the plasma membrane, enabling the cell as a whole to respond to temperature changes in its own external environment. This establishes a set of criteria for identifying the mitochondrial temperature sensor: it should be present in the OMM, probably with a homolog in the plasma membrane—or represented there by an analogous machinery; there should be clear homologs in almost all bacteria and it should be structurally and functionally related to previously characterized temperature sensing machinery in *B. subtilis*. The key component of the latter is the histidine kinase DesK, originally identified as a regulator of Δ5 acyl lipid desaturase and thus of membrane fluidity. Histidine kinases are widespread in bacteria, where they function in two‐component signaling systems, the histidine kinase moiety being embedded in the membrane, while the corresponding response regulator is located in the cytoplasm. Histidine kinases are fewer in number in eukaryotes [26] but, where present [27], act as sensors and regulators of osmolarity and extracellular chemical signals. DesK itself has clear homologs in gram‐negative bacteria, including *Escherichia coli* and the endosymbiotic *Rickettsia*, as well as in many fungi. Metazoan homologs are too diverged to ascribe orthology, and some have been annotated as sensors of other moieties, such as oxygen or nitrate. DesK itself contains multiple transmembrane segments and undergoes a temperature‐dependent conformational shift that activates signal transmission [25]. Some histidine kinase‐related signaling systems have been identified in mitochondria, e.g., the stress‐response regulator Srr1 of *Candida albicans* [28], or some isoforms of the NM23 family [29]. However, a specific link to mitochondrial temperature sensing is lacking. Intriguingly, one of the first reports of phospho‐histidine in eukaryotes was in bovine liver mitochondria [30], where the phosphorylated protein was later shown to be the key TCA cycle enzyme succinyl‐CoA synthase [31].

Approaching the issue from a completely different angle, if filamentation of the mitochondrial network is associated with temperature regulation—see later section on ‘Mitochondrial Dynamics’—one target of the temperature sensing machinery may also be an effector of mitochondrial fragmentation and/or hyperfusion. A possible candidate is the IMM scaffold protein SLP‐2, which regulates the balance between mitochondrial fission and fusion under different stress conditions. It also serves as a hub for integrating mitochondrial proteolysis, interacts with the machineries of mitophagic and apoptotic signaling and promotes OXPHOS activity at various levels [32, 33, 34].

Another possibly relevant observation from the bacterial world is the fact that many quorum‐sensing proteins [35]—surface proteins that limit bacterial growth in response to signals emitted by nearby cells, are temperature dependent. This suggests the possibility that cell density may be finely regulated to prevent overheating in a given environment. Quorum‐sensing proteins provide an alternative possible template for intracellular heat sensors in eukaryotes, raising the possibility that mitochondria might signal their temperature to the nucleus and other organelles by releasing molecules akin to bacterial quorum indicator molecules, such as homoserine lactones. Receptors for these molecules have been detected in human cells [36], where they are known to function as heat‐gated calcium channels and receptors for capsaicin, the ‘hot’ component of red chili peppers.

## Intracellular temperature gradients

A major question concerns the nature of temperature gradients between mitochondria and the rest of the cell, as well as between the various submitochondrial compartments. Despite the title of this article, it is well established that mitochondria are not uniform, rugby ball‐shaped entities floating in a similarly homogeneous cytosolic ‘ocean’. They are morphologically heterogeneous, adopting diverse shapes and internal structures, with many different arrangements of the inner membrane cristae, in which the OXPHOS enzyme complexes are embedded, seen in different organisms, cell types, or physiological conditions [37].

The surrounding cytoplasm is itself a protein‐rich environment containing a host of other organelles and membranous structures performing specialized physiological functions. Complex molecular assemblies link the inner and outer mitochondrial membranes (IMM, OMM) and endoplasmic reticulum (ER) [37, 38] with diverse endocytic, secretory and biosynthetic vesicles transiting between compartments [39, 40]. Thus, it is incorrect to imagine a sharp and invariant temperature boundary between mitochondria and the rest of the cell. Previous authors have not considered this morphological complexity when asserting that thermodynamic considerations preclude a significant temperature difference between mitochondria and cytosol, envisaged as two closely juxtaposed compartments [41]. Others have invoked ingenious mechanisms to account for sharp temperature differences between mitochondria and the extramitochondrial environment which would nevertheless be limited to approximately 7 °C [4, 42]. While we are not theoretical physicists, we see no reason to assume sharp temperature boundaries inside the cell that might challenge the laws of physics. Inside living cells, mitochondria adopt a variety of shapes, sizes and internal architecture, which are considered to reflect variations in physiological conditions, metabolic needs, and functional differentiation. Logically, therefore, there need not be a single or sharp boundary between hot mitochondria and cool cytosol in the cell. Rather, we argue that the various subcompartments are likely to be at intermediate temperatures, including some where a temperature gradient may exist over a much broader region than just a single phospholipid bilayer. Quantitation that we and others have been able to derive merely generates an average temperature of many mitochondria across many cells, although single‐cell imaging reveals heterogeneities both within and between cells [2, 43].

Using fluorescent probes, proteins or alternative methods, other authors have found evidence for clear temperature heterogeneities within cells [44, 45], with the nucleus typically warmer than the cytosol by at least 1 °C, and the temperature of mitochondria and other organelles influenced by physiological signals [46] or drug treatment [47, 48]. The low thermal diffusivity of the intracellular environment that has been inferred from the use of fluorescent probes [49] may account for these heterogeneous temperature distributions. Of note, attempts to reproduce the findings on mitochondrial temperature using isolated mitochondrial suspensions have not yet succeeded—or at least no such reports have been published, supporting the idea that the immediate cytosolic environment of mitochondria may be as important as processes within the organelle, in maintaining its internal temperature. There are also questions to be addressed, regarding the kinetics of observed fluorescence changes.

**Fig. 2:**
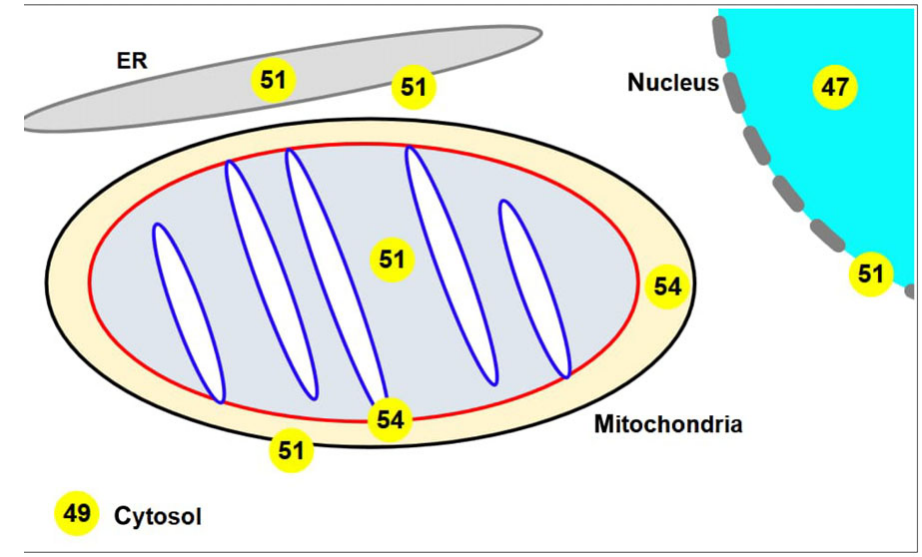
Meltome analysis of human proteins. Mean *T*_m_ (melting temperature, °C to nearest degree, shown in yellow circles) of proteins assigned to different subcellular compartments, based on the analysis presented in Fig. S1. Gray line: ER (endoplasmic reticulum) membrane, gray oval: ER lumen, turquoise circle: nucleoplasm, bold gray dotted line: nuclear envelope, white background: cytosol. Other symbols as in Fig. 1C.

The concept of heat gradients even between submitochondrial compartments is supported by meltome analysis [50] of human proteins. Those of the OXPHOS system have a *T*_m_ in the range 55–62 °C [51], some 5 °C higher than for mitochondrial proteins as a whole. A more granular analysis using the same method (Fig. 2, Fig. S1) indicates a clear gradation of *T*_m_ between the proteins of different sub‐mitochondrial and other cellular compartments: IMM ~ intermembrane space (IMS) > mitochondrial matrix > ER > OMM > cytosol > nucleus and other ‘mitochondrially distant’ compartments. Note that the *T*_m_ estimates for membrane‐bound proteins are almost certainly an under‐estimate, since the underlying method relies on the temperature profile for the precipitation of proteins deprived of their normal phospholipid environment, which will likely aggregate via unshielded hydrophobic domains.

As well as developing probes that can report the actual temperature of different subcellular compartments [49, 52], we need to consider the mechanisms of heat transfer between them. The relevant intracellular architecture comprises mitochondrial sub‐compartments that contain a plethora of unevenly distributed and oriented heating devices, such as ATPase nanomotors, while temperature heterogeneities likely exist within the cytosol too, as well as between it and other organelles. We postulate that an elaborate machinery must exist for monitoring the temperature of these many intracellular components and adjusting heat flow homeostatically, to prevent potentially catastrophic deviations. We know nothing of this machinery, beyond the existence of the heat‐shock proteins, which undoubtedly play a key role in protecting mitochondria from heat stress, as evidenced by the translocation of cytosolic heat‐shock proteins into the IMS after heat‐shock [53].

It is likely that heat conductance across membranes is influenced by phospholipid composition, by the proximity of closely juxtaposed membranes such as at contact sites between the OMM and IMM or at the mitochondria‐associated membranes (MAMs), where the OMM and ER interact and exchange components. With the ER itself constituting an interactive network, with bidirectional flow of materials into and out of the Golgi coupled to vesicle transport to and from the plasma membrane, heat transfer within the cell should be considered an active process, potentially susceptible to physiological regulation and thermal signaling, as envisaged by Okabe and Uchiyama [54].

Much current interest centers around liquid phase separations within cells [55], created by self‐organizing macromolecules and constituting barriers to the free movement of cellular materials. The underlying processes are temperature dependent, and should also affect heat conduction inside the cell. Thus, they will influence and be influenced by heat production and propagation from mitochondria, in ways that are currently unknown, but could be drastic in terms of cellular organization and function. Intracellular energy insufficiency, i.e., inadequate ATP supply, appears to promote exactly such a reorganization [56], with major cellular components sequestered into discrete compartments that can have pathological outcomes, if prolonged. But normal physiological events such as spindle formation during cell division also depend on temperature‐sensitive phase transitions [57].

The relationship between the temperature of mitochondria and that of the cytosol or plasma membrane may not be the same in all cell types or under all physiological conditions. Heat supply to cells in relative proximity to the external environment, e.g. skin fibroblasts, may need to be increased in the cold, which could be reflected in a slightly lowered mitochondrial temperature reflecting increased heat conduction to the rest of the cell. Organs that produce large amounts of heat due to their normal physiological functions, such as the heart, may also exhibit fluctuations in mitochondrial temperature indicative of homeostatic mechanisms operating at tissue or organ level.

## Mitochondrial dynamics

Despite over 30 years of intensive research, the physiological significance of the processes of mitochondrial fission and fusion remains incompletely understood. A widely accepted view is that the fission/fusion cycle, which occurs in most if not all cells, is a mechanism of mitochondrial quality control, enabling defective segments of the mitochondrial network to be identified after detachment, and routed for turnover by mitophagy [58]. This view is consistent with the observation that some metabolic stresses that induce mitochondrial dysfunction also lead to fission and mitophagy [59]. However, some other stresses that also result in a global dysfunction of mitochondria induce the opposite process, mitochondrial hyperfusion, which has been interpreted as a mechanism to resist mitophagy and thus protect mitochondrial integrity. The importance of the cycle has been vividly demonstrated in mice, where disruption of mitochondrial fission leads to a fatal cardiomyopathy, which can be compensated by a ‘reciprocal’ disruption of mitochondrial fusion [59]. The balance between fission and fusion appears to be set tissue‐specifically [59].

We here consider a different view, namely that fusion and fission, by altering the volume‐to‐surface area ratio of mitochondria, might modulate heat output from the organelle, while maintaining a constant intramitochondrial temperature. In other words, fragmented mitochondria, with a greater surface area for the same mitochondrial volume, should dissipate heat more effectively than tubular mitochondria. Fragmentation would therefore be predicted to be a response to increased heat production. In support of this idea, treatment of cells with an uncoupler such as FCCP, which leads to increased flux in the respiratory chain and an increase in mitochondrial temperature [47], entrains global fragmentation of mitochondria [60] and enhanced mitophagy [61]. In cells where mitochondrial fission or fusion is suppressed, mitochondria with low membrane potential are selectively marked for turnover [62]. The idea of mitochondrial fragmentation as a consequence of overheating, versus the widely accepted concept of its role in mitochondrial quality control, can be reconciled by considering sustained overheating to be a signature of the loss of electrochemical integrity of the inner mitochondrial membrane, due to irreversible damage to the OXPHOS machinery. Mitochondria also fragment in response to glucose starvation [63], which shifts the balance of cellular ATP production towards OXPHOS, which would also be predicted to lead to increased mitochondrial heat production. Mitochondrial fragmentation is also a response to externally applied heat stress in zebrafish [64], consistent with the proposition that fragmentation assists heat dissipation to prevent mitochondrial overheating.

Mitochondria where one of the OXPHOS complexes is non‐functional, but without loss of membrane integrity, should become cold rather than hot. The constitutive fission‐fusion cycle would still detach such mitochondria from the network. An extended version of our proposal is therefore that signaling from an as yet unknown sensor of mitochondrial heat production, for which the temperature of the outer mitochondrial membrane may be a marker, is what earmarks mitochondria for turnover. ‘Too hot’ or ‘too cold’ are both indicative of dysfunction and may each trigger a mitophagic response.

Mitochondrial hyperfusion can be considered a response to an external stress or signal that downregulates OXPHOS in the whole cell, even though the OXPHOS complexes themselves remain functional. It would serve the double purpose of minimizing heat conduction out of mitochondria while preventing the turnover of mitochondria detached from the network, due to an inappropriate ‘too cold’ signal. The exact conditions under which mitochondrial hyperfusion occurs are diverse. Amino‐acid starvation is one, but the metabolic imbalances produced by supplementation with specific amino acids actually enhance rather than relieve it [65]. Hyperfusion can also result from UV irradiation or treatment of cells with global inhibitors of macromolecular synthesis, such as actinomycin D or chloramphenicol [32]. Such treatments are expected to result in a large decrease in cellular ATP demand, with consequent and reported increases in cellular ATP level [32], which should inhibit OXPHOS and lead to decreased mitochondrial heat production. Since mitochondrial temperature is maintained under such conditions [2], it is logical to infer that heat diffusion from mitochondria should have been curtailed.

High‐resolution imaging, combined with mitochondrial temperature reporters, should now enable the relationship between mitochondrial ultrastructure and temperature to be explored in detail, and under different physiological conditions.

## Mitochondrial motility

In addition to their participation in a morphologically dynamic network, mitochondria move around inside the cell, using the cytoskeleton and motor‐protein complexes [66]. The physiological significance of this motility is debated and has been suggested to be based on chemotactic signals that induce mitochondria to move to where they are needed, in order to deliver ATP or other metabolites, or to return to the cell body, especially of neurons, where they collect materials needed to replenish their own bioenergetic machinery. However, their possible role as ‘portable heaters’ has been overlooked. If a region of the cell becomes too warm or too cold, ‘thermotaxis’ of active mitochondria should restore appropriate temperature. We posit that this should be important in large cells such as neurons, where mitochondrial trafficking to synapses may be essential for maintaining their temperature, a parameter likely to be essential for their diverse functions, i.e., neurotransmitter synthesis, release and reuptake. Axonal mitochondria may also be essential to maintaining a steady temperature along the length of axons, for which the surrounding myelin sheath may provide thermal as well as electrical insulation [67]. The ability of mitochondria to detect and correct thermal anomalies by moving within cells should also be functionally important in other large cells, such as megakaryocytes and oocytes.

Once again, the availability of diverse intracellular temperature reporters should enable the hypothesis that mitochondria do move, or can be induced to move, towards cool regions of cells in order to deliver heat, to be directly tested.

## Microbes, fever and immunity

We have proposed that high mitochondrial temperature is a natural and unavoidable consequence of OXPHOS activity. Since aerobic bacteria, from which the mitochondrial ancestor arose, perform a similar chemistry, it is a reasonable assumption that they also produce substantial amounts of heat, compared, for example, with obligate anaerobes. Moreover, having adapted to life in comparatively cool environments, such as soil, water and even the bodies of mammals, they must either have evolved ways of rapidly dissipating this heat, with their proteins adapting progressively so as to function at lower temperatures, or else retaining much of the heat internally, enabling their proteins to continue to operate at the same high temperature as in mitochondria. On the first model, the bacterial cell wall would represent a conducting layer, on the second model an insulating layer.

Meltome analysis of *E. coli* proteins [68] revealed that the *T*_m_ of typical ‘cytosolic’ proteins was around 56 °C, and reaching well above 60 °C for many proteins of the inner and outer membranes and the periplasmic space. This is consistent with the respiratory membrane being the major source of heat, and the cell wall providing insulation, although bacterial cell walls have been postulated to have numerous other, additional functions. Intriguingly, metabolic activity in bacteria induces a substantial change in the fluidity of the cytoplasm, transforming its properties from solid‐ to liquid‐like, and enabling the size‐dependent motion of intracellular components that is essential for diverse biological processes [69]. This strongly supports the idea that bacterial heat production under metabolically active conditions sustains vital dynamic processes, while vitrification of the cytoplasm during metabolic dormancy helps to maintain cellular architecture. The intracellular temperature of bacteria can now be profiled directly, using similar fluorescent probes and reporters as have been successfully employed in eukaryotic cells.

Heat may also have a role in enabling animals to resist or eliminate infection by pathogens. Fever is a common feature of infectious diseases, and has generally been interpreted as an indicator of an active immune response. The physiological purpose of a 2–3 °C increase in body temperature has been extensively debated, but the consensus is that it enables or enhances a variety of immunity‐related processes [70]. It should be noted, however, taking *E. coli* as an example, that a small increase in external temperature has little effect on bacterial growth [71], and for some enteric bacteria may even stimulate it [72], while the survival of at least some strains of *E. coli* is far less impaired at high temperatures—even up to 53 °C [73] than human cells, which are unable to tolerate a sustained environmental temperature in excess of about 43 °C [74].

Given the threat to life posed by pathogens, and the threat to pathogens from the immune system, it is logical that multiple strategies have evolved on both sides in the evolutionary arms race. Mitochondria have been implicated as primary sources of reactive oxygen species (ROS), which can act as agents or at least as signal initiators of cell killing by cytotoxic T‐cells or NK cells, or inside phagocytes. Establishment of a pro‐inflammatory state in macrophages has been postulated to involve a reprogramming of mitochondrial metabolism to favor reverse electron flow through OXPHOS complex I [75]. There is an ever‐expanding literature on the undoubtedly diverse roles of mitochondria in immunity, far beyond the scope of this review. However, the direct delivery of heat by immune cells, either as a signal or even as a cytotoxic agent, has been overlooked. It could at least partially underlie the phenomenon of fever, which would be a systemic signature of local heat induction at sites of infection and pathogen killing.

## OXPHOS diseases

The realization that heat production is a core process of mitochondria, and that mitochondrial components—both proteins and nucleic acids, function at elevated temperature, leads to the conclusion that defects in the OXPHOS machinery can lead to temperature anomalies with potentially pathological consequences. Mitochondrial diseases have been recognized for over half a century, but have mainly been considered as consequences of insufficient ATP production, oxidative stress due to excessive ROS or metabolic derangement resulting from interference with the TCA cycle and/or other elements of secondary metabolism. However, we need to consider mitochondrial heat production as a further and possibly major contributor to the pathology of OXPHOS diseases.

Indeed the very first such disease to be recognized and studied, nowadays known as Luft's disease after its discoverer, had chronic hyperthermia and hypermetabolism as cardinal features [76], reflecting partial uncoupling of OXPHOS [77] and associated with structural anomalies in the cristae [78]. However, a more general interference with mitochondrial heat output or temperature as a consequence of OXPHOS mutations has received little attention. Nevertheless, some classic features of OXPHOS diseases suggest that anomalies in heat production could play a part in the pathogenic process. These include hypothermia in the brain [79] as well as episodes of systemic hypothermia [80, 81] and isolated reports of hyperthermia [82, 83]. The pathological features of OXPHOS diseases are diverse, complex, and underlying mechanisms are still debated. We propose that defective mitochondrial thermoregulation should be considered as a possible contributory factor: homeostatic maintenance of mitochondrial temperature in cases of OXPHOS deficiency may lead to other metabolic consequences, such as futile ATP hydrolysis, metabolite imbalances or a decrease in heat conductance to the rest of the cell. In turn, the latter may impact diverse processes, including the action of membrane transporters, cytoskeletal dynamics, vesicle trafficking, protein synthesis, and gene expression. Importantly, whereas we and others [84] have focused on the effects of various cellular treatments on the temperature of the mitochondria, it may be more appropriate to study the effects of aberrant mitochondrial heat output on the temperature and metabolism of other cellular compartments. A relevant example is the recent finding that mitochondrial heat production activates neural excitation in response to a calcium signal, via a rapidly generated 2 °C rise in cytosolic temperature [85].

The deposition of protein aggregates, principally of α‐synuclein in the form of Lewy bodies in cases of neurodegeneration, has been proposed to be associated with mitochondrial dysfunction [86]. A plausible molecular mechanism would involve a deficiency of mitochondrial heat production, which would normally resolve these aggregates or prevent their formation. The thermogenic nature of the alternative oxidase may thus underlie its ability to alleviate the pathological effects of neuronal expression in *Drosophila* of the human beta‐amyloid peptide associated with Alzheimer's disease [87], as well as developmental defects in cell migration due to disrupted steroid [88] or JNK signaling [89]. In each of these cases, we hypothesize that increased heat output from mitochondria can resolve proteotoxic stress or cell migration defects arising from other causes. Cell migration has elsewhere been shown to be stimulated by elevated temperature [90].

We suggest that deranged mitochondrial heat production or diffusion should be considered as possible pathogenic mechanisms, while measures to facilitate heat delivery or its dissipation deserve consideration as plausible therapeutic strategies.

## Conclusion

The issue of heat production in mitochondria and its physiological impact have been largely overlooked. In addition to the points raised here, many other topics in thermobiology and medicine, together with their mitochondrial dimension, remain to be explored. For example, obesity provides protective insulation against cold stress in humans, yet shows no correlation with ambient temperature [91] and the mitochondria of cancer cells are variously reported to be either at a higher or lower temperature than those of control cells [92]. How mitochondrially produced heat is dissipated and buffered in cells remain major unresolved questions, highlighted by the fact that cytosolic and plasma membrane temperatures fluctuate only minimally, despite large metabolic changes [93]. The fact that mitochondria are functionally disabled when subjected to externally applied heat additional to that produced endogenously [24], indicates that these questions are as important as how mitochondrial heat production is itself regulated. We have much still to learn.

## Conflict of interest

The authors declare no conflict of interest.

## Author contributions

HTJ, MT, PB, and PR jointly conceived the contents of this paper. DD (Fig. 1, Fig. S1) and MT (Fig. 2A,B) designed and conducted the analyses described. HTJ drafted the figures and manuscript which were reviewed and edited by all authors.

## Supporting information


**Fig. S1.** Meta‐analysis of protein thermal stabilities at suborganellar resolution. For relevant methods, see references [94, 95, 96], as cited in the legend for Figure S1.
**Table S1.** Source data for Fig. 1.

## Data Availability

All data are included in the paper, including Supporting Information (for source data see Table S1).
