# Supplementary material for: Mitochondria: great balls of fire
Source: FEBS J. 2024 Nov 14;291(24):5327–41. doi: 10.1111/febs.17316 (PMC11653699; doi:10.1111/febs.17316)
Supplement: Supplementary file 1 — Fig. S1. Meta‐analysis of protein thermal stabilities at suborganellar resolution. For relevant methods, see references [94, 95, 96], as cited in the legend for Figure S1. Table S1. Source data for Fig. 1. [file FEBS-291-5327-s001.pdf]

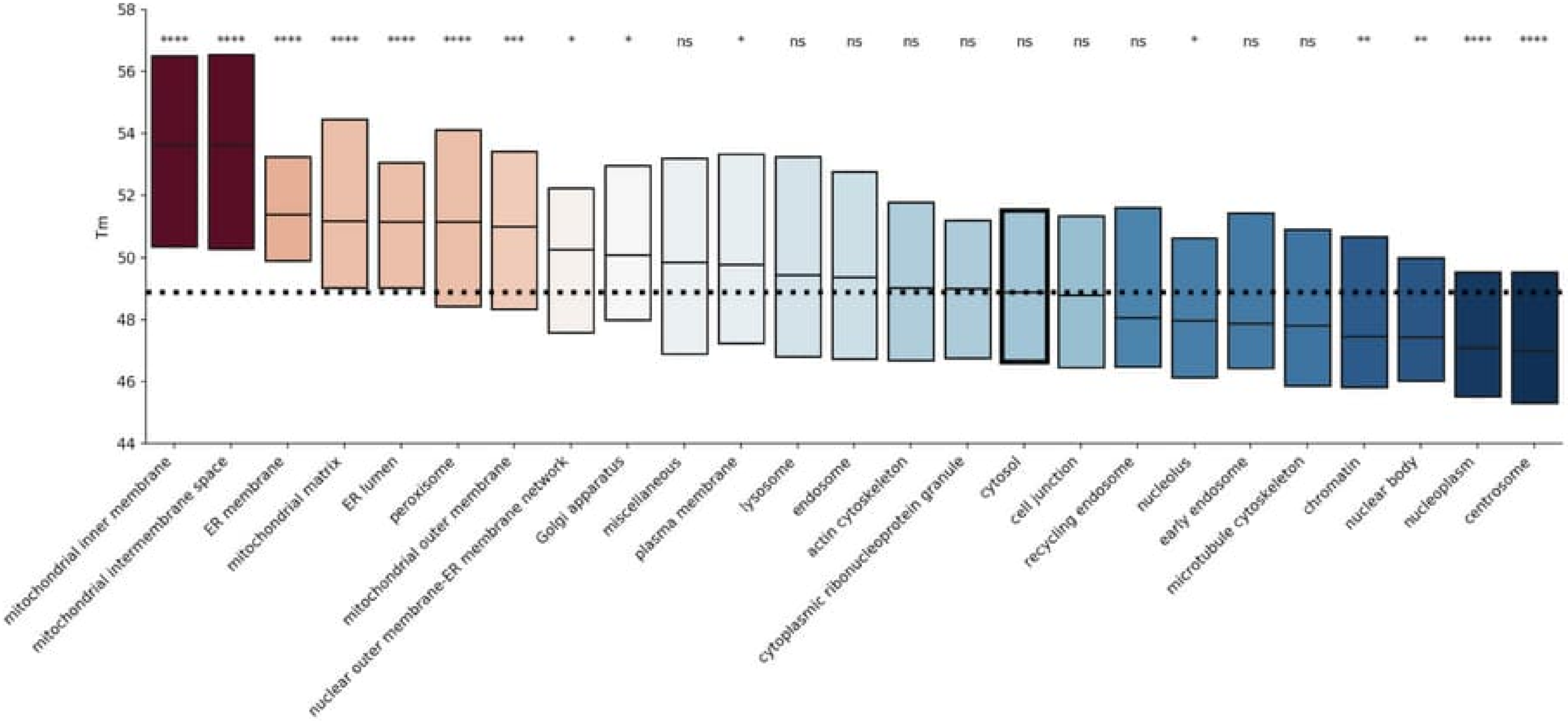

Supplementary Figure S1

Meta-analysis of protein thermal stabilities at suborganellar resolution. Thermal proteome profiling data of intact K562 and Jurkat human cell lines [94, 95] were crossed with data on locations of proteins in human cells, derived from proximity biotinylation coupled with mass spectrometry [96]. Box plots indicate median (bars) and IQR for each subcellular location. All submitochondrial locations, i.e. the IMS, IMM, OMM, and matrix are enriched for relatively thermostable proteins, with median Tm values ranging between 51-54 ºC. The median Tm of all proteins considered (dotted line) is 49 ºC. Box plots are sorted by median values and coloured from least thermostable (dark blue) to most thermostable (dark red). For statistical significance determination, the distribution of Tm values for each location was compared to Tm values across the entire proteome, as indicated by the p-values along the y-axis (Wilcoxon rank-sums test – *, **, *** and **** corresponding to $p < 0.05$, 0.01, 0.001, 0.0001 respectively. ns – non-significant, $p > 0.05$). Only locations with >10 data points are shown. Some proteins appear in multiple locations.

Supplementary Table S1

Source data for Figure 1: mitochondrial temperature shifts estimated by MTY fluorescence, as described by Terzioglu et al., 2023 [2], in ºC.

A

COLD SHOCK (37→31)

	initial drop	peak cooling	final
	3.0	13.6	3.6
	5.6	16.7	4.8
	2.1	14.6	-2.0
	3.4	15.0	2.3
	4.2	12.5	2.8
mean	3.7	14.5	2.3
stdev	1.3	1.6	2.6
n =	5	5	5

B

HEAT SHOCK (37→40)

	initial rise	peak cooling	peak warming	final
	1.6	10.5	5.1	7.6
	0.9	7.5	0.7	6.3
	-0.3	11.2	0.8	10.5
mean	0.7	9.7	2.2	8.1
stdev	1.0	2.0	2.5	2.2
n =	3	3	3	3.0
